# Sterbehilfe international und die Debatte in Deutschland

**DOI:** 10.1007/s00103-026-04189-8

**Published:** 2026-02-23

**Authors:** Ute Lewitzka, Christian Petzold

**Affiliations:** 1 Professur für Suizidologie und Suizidprävention, Zentrum für Psychische Gesundheit, Klinik für Psychiatrie, Psychosomatik und Psychotherapie Universitätsklinikum Frankfurt, Goethe Universität, Heinrich-Hoffmann-Str. 10, 60528 Frankfurt, Hessen Deutschland; 2 Klinik und Poliklinik für Psychiatrie und Psychotherapie, Universitätsklinikum Carl Gustav Carus, TU Dresden, Dresden, Sachsen Deutschland; 3 Werner-Felber-Institut für Suizidprävention und interdisziplinäre Forschung im Gesundheitswesen e. V., Dresden, Sachsen Deutschland; 4 Bundesärztekammer, Berlin, Deutschland

**Keywords:** Suizidassistenz, Sterbehilfe, Euthanasie, Rechtliche Rahmenbedingungen, Gesellschaftliche Auswirkungen, Assisted suicide, Euthanasia, Legal framework, Social implications

## Abstract

Der Artikel gibt einen Überblick über rechtliche, politische und gesellschaftliche Rahmenbedingungen und Entwicklungen der Sterbehilfe in ausgewählten Ländern. Dabei zeigt sich, dass die Sterbehilfe in vielen Ländern zunächst an bestimmte, eng gefasste Bedingungen (z. B. unheilbare oder terminale Erkrankungen) geknüpft war und innerhalb eines relativ kurzen Zeitraums sowohl hinsichtlich der rechtlichen Zugangsvoraussetzungen als auch der praktischen Anwendung ausgeweitet wurde. In Ländern wie den Niederlanden oder Kanada war von Beginn an auch die Tötung auf Verlangen zugelassen, die dort den weitaus größten Teil der dokumentierten Fälle ausmacht.

Im Gegensatz zu den beschriebenen internationalen Entwicklungen befindet sich Deutschland nach dem Urteil des Bundesverfassungsgerichts vom 26.02.2020 in einem Prozess der gesetzlichen und gesellschaftlichen Neuorientierung. Das Urteil des Bundesverfassungsgerichts (BVerfG) erkennt das Recht auf selbstbestimmtes Sterben (wie zuvor das Bundesverwaltungsgericht (BVerwG)) als Ausdruck von Autonomie (allgem. Persönlichkeitsrecht – Art. 1 Abs. 1, 2 Abs. 1 GG) verbindlich an, jedoch fehlen bislang prozedurale und institutionelle Rahmenbedingungen, die eine gesetzlich geregelte Umsetzung ermöglichen. So existiert z. B. kein verbindliches Verfahren zur Feststellung der Freiverantwortlichkeit.

Eine Gegenüberstellung der Entwicklung in Deutschland mit anderen Ländern ist nicht möglich, da es keine separate Statistik zu den Suizidassistenzzahlen gibt, d. h., „reguläre“ Suizide werden gemeinsam mit Suizidassistenzen in einer Kategorie der ICD-10 erfasst und vom statistischen Bundesamt veröffentlicht. Die bekanntgemachten Zahlen der Sterbehilfeorganisationen werden nicht geprüft, zeigen jedoch eine deutliche Zunahme der Suizidassistenzen seit 2020.

## Abstract (en)

The article provides an overview of the legal, political, and social framework conditions and developments relating to euthanasia in selected countries. It has been demonstrated that the practice of euthanasia was initially subject to certain narrowly defined conditions (e.g., incurable or terminal illnesses) in many countries and was expanded within a relatively short period of time, both in terms of legal access requirements and practical application. In countries such as the Netherlands and Canada, active euthanasia was also permitted from the outset and accounts for the vast majority of documented cases there.

In contrast to the international developments described above, Germany is still in the process of legal and social reorientation following the 2020 ruling of the Federal Constitutional Court. Despite the ruling’s establishment of the fundamental admissibility of assisted suicide, a paucity of procedural and institutional regulations exists to facilitate uniform practice.

At this time, a comparison of developments in Germany with those in other countries is not possible, due to the absence of separate statistics on assisted suicide cases. The Federal Statistical Office continues to record and report both “regular” and assisted suicides in the same category of the International Classification of Diseases, Tenth Revision (ICD-10). The figures published by euthanasia organizations have not been officially verified, but they do indicate a significant increase in documented cases since 2020.

## Einleitung

In der internationalen wissenschaftlichen Literatur wird der Begriff Euthanasia häufig als Oberbegriff für verschiedene Formen der Lebensbeendigung auf Verlangen oder der Suizidassistenz verwendet. Im deutschsprachigen Raum wird hingegen differenziert zwischen rechtlich und ethisch unterschiedlich bewerteten Formen der Sterbehilfe. Die hier verwendete Terminologie folgt den Definitionen und Empfehlungen des Deutschen Ethikrats [2] sowie gängigen medizin- und rechtsethischen Abgrenzungen.

*Sterbenlassen (passive Sterbehilfe)* bezeichnet den Verzicht auf oder das Abbrechen lebensverlängernder Maßnahmen, wenn dies dem erklärten oder mutmaßlichen Willen der betroffenen Person entspricht. Sie ist rechtlich zulässig, sofern keine medizinische Indikation mehr besteht und der Patientenwille dokumentiert ist, wobei sowohl das Fehlen der medizinischen Indikation als auch ein entgegenstehender, konkret auf die Ablehnung der jeweiligen Therapie gerichteter Patientenwille jeweils für sich das Sterbenlassen begründen können. Eine* Therapie am Lebensende (indirekte Sterbehilfe)* beinhaltet z. B. die Gabe potenziell lebensverkürzender Medikamente (z. B. hochdosierter Schmerzmittel), wenn die Linderung von Leiden im Vordergrund steht und eine mögliche Lebensverkürzung lediglich als unbeabsichtigte Nebenfolge in Kauf genommen wird. Auch diese Form ist erlaubt, sofern die Maßnahme medizinisch indiziert ist und dem Willen der betroffenen Person entspricht.

*Tötung auf Verlangen* bezeichnet die gezielte und unmittelbare Herbeiführung des Todes eines Menschen durch eine andere Person – etwa durch die Verabreichung eines tödlich wirkenden Mittels – auf ausdrückliches und ernstliches Verlangen des Betroffenen. Diese Handlung ist in Deutschland gemäß § 216 Strafgesetzbuch (StGB) verboten [1], selbst wenn sie auf Wunsch der sterbewilligen Person erfolgt.

Der* (ärztlich) assistierte Suizid* stellt demgegenüber eine Form der straflosen Beihilfe zur Selbsttötung dar. Ärzt:innen dürfen hierbei die Vorbereitung einer eigenverantwortlichen Selbsttötung unterstützen – etwa durch das Verschreiben oder Bereitstellen eines tödlich wirkenden Mittels –, jedoch nicht durch dessen aktive Verabreichung. Nach dem Urteil des Bundesverfassungsgerichts von 2020 (BVerfGE 153, 182) ist der assistierte Suizid in Deutschland grundsätzlich zulässig, sofern die Entscheidung freiverantwortlich und wohlerwogen getroffen wird.

Die Debatte um die Sterbehilfe in Deutschland befördert häufig grundsätzliche Fragen zum Lebensende: Wer darf selbst über sein Lebensende bestimmen? Ab wann gilt ein Leben als nicht mehr lebenswert? Und wer trägt die Verantwortung für die Entscheidung in einer Zeit, in der die Interventionsmöglichkeiten der Medizin das Handlungsspektrum immer weiter ausdehnen und das Sterben zunehmend hinauszögern können, in der gesellschaftliche Werte sich rasant wandeln und das Lebensende zunehmend gestaltbar, ja zur Planungsaufgabe wird [3]? Der Tod ist heute nicht mehr nur ein natürlicher oder ein religiös geprägter Prozess – er ist auch oftmals ein juristischer, medizinischer und persönlicher Akt.

Freiverantwortliche Suizidentscheidungen sind ethisch wie rechtlich zu respektieren [2]. Das Bundesverfassungsgericht hat 2020 das Recht auf ein selbstbestimmtes Sterben [4] als Ausdruck des allgemeinen Persönlichkeitsrechts bestätigt. Es betont, dass eine Suizidentscheidung nur dann als *freiverantwortlich* gilt, wenn sie auf einem eigenständigen, wohlerwogenen und dauerhaften Willen beruht und nicht durch Druck, Täuschung oder akute psychische Krisen beeinflusst ist [5].

Rechtliche Rahmenbedingungen und die sich damit eröffnenden Möglichkeiten, „das Sterben zu beschleunigen“, können Einstellungen, Haltungen und möglicherweise Handlungsweisen sowohl des Einzelnen als auch einer Gesellschaft verändern und prägen. Die Auseinandersetzung mit dem Thema macht deutlich, dass es um einen Balanceakt zwischen individueller Autonomie und gemeinsamen Normen geht – und um die Werte, die unser Miteinander prägen [6].

In den vergangenen Jahrzehnten haben mehrere Staaten unterschiedliche Regelungsmodelle für die (ärztliche) Suizidassistenz und die Tötung auf Verlangen entwickelt. Dabei zeigen sich deutliche Unterschiede hinsichtlich der rechtlichen Voraussetzungen, der praktischen Umsetzung und der gesellschaftlichen Akzeptanz. Während in einigen Ländern – etwa in den Benelux-Staaten oder Kanada – eine Liberalisierung mit breitem Zugang erfolgt ist, bestehen in anderen Ländern weiterhin restriktive Rahmenbedingungen. Diese Vielfalt an Modellen bietet die Möglichkeit, internationale Erfahrungen zu vergleichen und daraus Erkenntnisse für zukünftige Regelungen zu gewinnen [7]. Die Gründe für die unterschiedlichen Entwicklungen in den Ländern sind vielfältig. Neben kulturellen und religiösen Faktoren spielen politische Mehrheiten, rechtliche Traditionen und medizinethische Vorstellungen eine zentrale Rolle. Auch der wirtschaftliche Entwicklungsstand ist bedeutsam: In wohlhabenden Staaten bestehen meist ausgebaute Gesundheitssysteme, verankerte Patientenrechte und eine öffentliche Debattenkultur, die ethische, medizinische und juristische Perspektiven miteinander verknüpft. Dort steht häufig die individuelle Selbstbestimmung im Vordergrund.

In vielen Ländern mit geringerem Einkommen hingegen sind Tötung auf Verlangen und Suizidassistenz meist verboten – teils aus religiösen Gründen, teils weil die institutionellen und medizinischen Strukturen, einschließlich einer flächendeckenden Palliativversorgung, fehlen, um solche Verfahren sicher und ethisch verantwortbar zu gestalten [2, 8]. In Kolumbien und Ecuador – Ländern mit mittlerem Einkommen – haben Gerichtsentscheidungen liberalere Regelungen ermöglicht; ähnliche Entwicklungen zeichnen sich derzeit in Uruguay ab. In Kanada dominiert die Tötung auf Verlangen (Medical Assistance in Dying, MAID), während in den USA ausschließlich der ärztlich assistierte Suizid zulässig ist. In Europa bestehen weiterhin deutliche Unterschiede: Einige Staaten verfolgen restriktive Modelle, andere diskutieren oder erproben eine schrittweise Liberalisierung.

Die Haltung zur Sterbehilfe ist weltweit das Ergebnis eines komplexen Zusammenspiels aus wirtschaftlichen, kulturellen, politischen und ethischen Faktoren. Ein Verständnis der internationalen Debatte erfordert daher die Kenntnis dieser unterschiedlichen Rahmenbedingungen. Der folgende Beitrag vergleicht die rechtlichen Grundlagen und gesellschaftlichen Entwicklungen der Sterbehilfe in ausgewählten Ländern. Ziel ist es, aufzuzeigen, welche Regelungsmodelle sich herausgebildet haben und in welcher Weise rechtliche Strukturen, öffentliche Diskurse und kulturelle Wertvorstellungen deren Ausprägung bestimmt haben.

## Historischer Hintergrund und aktuelle Entwicklung ausgewählter Länder

Analysiert werden Länder, die unterschiedliche rechtliche und gesellschaftliche Zugänge zur Sterbehilfe repräsentieren. Dazu zählen die Niederlande und Belgien als Beispiele für frühzeitige Legalisierungen mit fortschreitender Ausweitung, die Schweiz mit ihrer einzigartigen Vereinsstruktur, Kanada als Beispiel einer rasanten Liberalisierung nach gerichtlicher Entscheidung sowie ausgewählte europäische Länder mit restriktiveren Regelungen. Die Einbeziehung Belgiens neben den Niederlanden erfolgt bewusst, da sich dort – trotz ähnlicher rechtlicher Grundlagen – eine besonders weitgehende gesellschaftliche Normalisierung, einschließlich der Zulassung aktiver Sterbehilfe bei Minderjährigen, beobachten lässt. Österreich wurde aufgrund des noch jungen Rechtsrahmens (seit 2022) nicht in die Analyse aufgenommen.

Die vorliegende Arbeit folgt dem Ansatz einer narrativen Übersicht. Grundlage der Darstellung sind nationale Gesetzestexte, Regierungsdokumente, einschlägige Gerichtsurteile sowie aktuelle Publikationen in wissenschaftlichen Fachzeitschriften (z. B. *Annals of Palliative Medicine, Journal of Medical Ethics, Deutsches Ärzteblatt*). Ergänzend wurden Berichte und Stellungnahmen offizieller Institutionen (z. B. nationale Ethikräte, Fachgesellschaften) sowie aktuelle Medienanalysen herangezogen, um die gesellschaftlichen Diskurse abzubilden.

Die Auswahl der Länder erfolgte nach dem Kriterium, unterschiedliche rechtliche Modelle und gesellschaftliche Kontexte der Suizidassistenz darzustellen. Die Darstellung konzentriert sich auf Entwicklungen seit den 2000er-Jahren, mit einem Fokus auf aktuelle Reformen und rechtliche Dynamiken bis 2025. Ziel ist nicht eine systematische Vollerhebung, sondern eine strukturierte Zusammenfassung zentraler Entwicklungen und Regelungsmodelle, die internationale Vergleichbarkeit ermöglichen.

### Schweiz

Die Schweiz war der erste Staat weltweit, der die Beihilfe zum Suizid unter bestimmten Bedingungen nicht mehr strafbar stellte. Bereits im 1918 publizierten Entwurf des Schweizerischen Strafgesetzbuches, das 1942 in Kraft trat, wurde in Artikel 115 festgelegt, dass Suizidassistenz nur dann strafbar ist, wenn sie aus eigennützigen Motiven erfolgt. Ursprünglich sollte diese Regelung Einzelfälle individueller Unterstützungshandlungen erfassen, nicht jedoch die heute institutionalisierte Form organisierter Sterbehilfe.

Seit den 1980er-Jahren prägen vor allem 2 Organisationen die Praxis der Suizidhilfe in der Schweiz: EXIT, die ausschließlich in der Schweiz wohnhafte Personen begleitet, und DIGNITAS, die auch Menschen aus dem Ausland unterstützt. Während EXIT die gesellschaftliche Wahrnehmung und Normalisierung der Suizidhilfe im Inland maßgeblich geprägt hat, konzentriert sich DIGNITAS auf Fälle sogenannter Sterbetouristen. Diese institutionalisierte Praxis macht die Schweiz zum einzigen Land weltweit, in dem Sterbetourismus rechtlich möglich ist. Nach Angaben von DIGNITAS stammte bis 2021 rund ein Drittel der begleiteten Personen aus Deutschland; erst seit 2022 ist diese Zahl rückläufig. Unter den ausländischen Sterbewilligen dominierten zuletzt Personen aus Frankreich, dem Vereinigten Königreich und Italien, was die anhaltend internationale Bedeutung der Schweizer Regelung verdeutlicht.

In den vergangenen Jahren ist in der Schweiz ein deutlicher Anstieg der Zahl begleiteter Suizide zu verzeichnen. Nach Angaben des Bundesamts für Statistik (BFS) stieg die Zahl der registrierten assistierten Suizide von 1176 im Jahr 2018 auf 1729 im Jahr 2023 – ein Zuwachs um rund 47 % innerhalb von 5 Jahren. Die Mehrzahl der Fälle betrifft ältere Personen, wobei Frauen leicht überrepräsentiert sind [9].

Neben der demografischen Alterung [10] und der Zunahme chronisch degenerativer Erkrankungen spielt auch eine wachsende gesellschaftliche Akzeptanz des assistiert-selbstbestimmten Sterbens eine zentrale Rolle. Parallel dazu formieren sich neue Akteursgruppen, die das bestehende Modell weiterentwickeln oder radikal verändern wollen. Exemplarisch hierfür steht die 2024 gegründete Menschenrechtsorganisation *The Last Resort *[11], die kostenfreie Suizidassistenz anbietet und sich ausschließlich über Spenden finanziert.

Besondere öffentliche und juristische Aufmerksamkeit erhielt *The Last Resort* durch den Einsatz der sogenannten Sarco-Kapsel, die vom australischen Aktivisten Philip Nitschke entwickelt wurde. Dieses mittels 3D-Druck hergestellte Gerät soll einen raschen Tod durch Einleitung von Stickstoff herbeiführen. Erstmals öffentlich vorgestellt wurde die Kapsel bereits 2018, international diskutiert wird sie jedoch vor allem seit ihrer Präsentation in der Schweiz im Juli 2024. Am 23.09.2024 erfolgte im Kanton Schaffhausen der erste dokumentierte assistierte Suizid mit dem „Sarco“. Bundesrätin Elisabeth Baume-Schneider bezeichnete den Einsatz noch am selben Tag im Nationalrat als „nicht rechtskonform“. Die Kapsel ist weder als Medizinprodukt zugelassen noch unterliegt Stickstoff als verwendetes Gas dem Heilmittelgesetz. Vertretende der Sterbehilfeorganisation EXIT äußerten sich zurückhaltend und betonten, dass die Methode für die in der Schweiz etablierte Praxis der Suizidhilfe derzeit keine Relevanz habe. Der Fall verdeutlicht bestehende rechtliche Grauzonen und unterstreicht den Bedarf an einer verbindlichen gesetzlichen Regelung der Suizidassistenz [12].

Die Praxis der Suizidassistenz in der Schweiz wird seit Jahrzehnten wesentlich durch zivilgesellschaftliche Organisationen mitgestaltet. Insbesondere EXIT hat durch seine langjährige Tätigkeit zur gesellschaftlichen Etablierung und Normalisierung der Thematik beigetragen. Zugleich kommt den standesethischen Leitlinien der Schweizerischen Akademie für Medizinische Wissenschaften (SAMW) in Ermangelung spezifischer gesetzlicher Regelungen eine besondere normative Bedeutung zu. Diese Leitlinien definieren die Bedingungen, unter denen Ärzt:innen Suizidhilfe leisten dürfen, und spiegeln den anhaltenden Diskurs zwischen einer traditionell zurückhaltenden ärztlichen Haltung und einer zunehmend liberalen gesellschaftlichen Praxis wider [13].

Ein markantes Beispiel ist die am 01.07.2023 in Kraft getretene Gesetzesänderung im Kanton Zürich (§ 38a Gesundheitsgesetz; [14]), die es Bewohnerinnen und Bewohnern öffentlich finanzierter Alters- und Pflegeheime ermöglicht, Sterbehilfe in den Einrichtungen selbst in Anspruch zu nehmen. Diese Reform geht zurück auf die Volksinitiative „Selbstbestimmung am Lebensende auch in Alters- und Pflegeheimen“, die maßgeblich von EXIT und weiteren zivilgesellschaftlichen Akteuren unterstützt wurde. Die ursprüngliche Initiative strebte eine Ausweitung der Duldungspflicht auf sämtliche Einrichtungen des Gesundheits- und Sozialwesens – einschließlich Spitälern, psychiatrischen Kliniken und Justizvollzugsanstalten [15] – an. Der Zürcher Regierungsrat bewertete diesen Ansatz als übermäßig weitreichend, unterstützte jedoch die Öffnung der Alters- und Pflegeheime. Das zentrale Argument der Befürworter lautet, dass Menschen dort sterben können sollten, wo sie ihre letzte Lebensphase verbringen – und nicht ausschließlich zu Hause oder in externen Sterbeeinrichtungen.

Diese strategische Vorgehensweise setzt sich in weiteren kantonalen Volksinitiativen fort, die rechtliche Verpflichtungen zur institutionellen Duldung von Sterbehilfe schaffen sollen. Kritiker warnen hierbei vor potenziellen psychischen Belastungen für Mitbewohnerinnen und Mitbewohner sowie das Pflegepersonal und vor möglichen Veränderungen des sozialen Klimas in den Einrichtungen. Gleichwohl verdeutlicht diese Entwicklung, dass Sterbehilfeorganisationen zunehmend als politische Akteure auftreten, um die gesellschaftliche und institutionelle Akzeptanz der Sterbehilfe zu verankern.

Die Schweizer Situation illustriert exemplarisch die Spannungsfelder im internationalen Diskurs: Einerseits ist das rechtliche Umfeld vergleichsweise liberal, andererseits bleiben die institutionellen Strukturen fragil. Während etablierte Organisationen medizinische Standards und ethische Leitlinien betonen, verfolgen neue Akteure eine Ausweitung der rechtlichen Grenzen mit dem Ziel, Sterbehilfe als universelles Menschenrecht zu verankern und technisch zu vereinfachen. Die Schweiz steht damit paradigmatisch für die komplexe Aushandlung zwischen individueller Autonomie, staatlicher Regulierung und ethischer Verantwortung im Kontext des assistierten Suizids.

### Niederlande

Die Niederlande gelten als internationaler Vorreiter in der gesetzlichen Regulierung der aktiven Sterbehilfe. Mit dem Inkrafttreten des „Gesetzes über die Kontrolle der Lebensbeendigung auf Verlangen und der Hilfe bei der Selbsttötung“ im Jahr 2002 wurde als weltweit erstes nationales Regelwerk die Tötung auf Verlangen unter klar definierten Voraussetzungen legalisiert. Die ärztliche Durchführung ist seither zulässig, sofern sämtliche gesetzlich normierten Sorgfaltskriterien erfüllt sind: Die Entscheidung der betroffenen Person muss freiwillig und nachweislich wohlüberlegt getroffen worden sein; das Leiden muss als unerträglich und ohne Aussicht auf Besserung eingestuft werden; es muss eine unabhängige ärztliche Zweitmeinung vorliegen und die Durchführung hat medizinisch fachgerecht zu erfolgen [16].

Eine Besonderheit des niederländischen Rechts ist die gesetzliche Zulässigkeit vorausschauender Verfügungen zur Sterbehilfe. Diese schriftlichen Erklärungen können auch dann umgesetzt werden, wenn die betroffene Person infolge fortgeschrittener Demenz oder irreversiblen Bewusstseinsverlusts nicht mehr in der Lage ist, ihren Willen zu äußern. Voraussetzung ist, dass die Erklärung in einem Zustand der Urteilsfähigkeit verfasst wurde, klar und eindeutig formuliert ist und die allgemeinen Sorgfaltskriterien eingehalten werden. Die abschließende Prüfung der Recht- und Ordnungsmäßigkeit obliegt den Regionalen Prüfungskommissionen für Sterbehilfe (*Regionale Toetsingscommissies Euthanasie*, RTE). Die RTE veröffentlichen jährlich detaillierte Berichte über die Anzahl, Art und Bewertung der gemeldeten Fälle und bilden damit das zentrale Kontrollorgan des niederländischen Systems [17].

Im Jahr 2016 führte eine niederländische Ärztin Sterbehilfe bei einer dementen Patientin durch, die Jahre zuvor eine schriftliche Verfügung verfasst hatte. Die Patientin war zum Zeitpunkt der Sterbehilfe nicht mehr urteilsfähig und zeigte während der Durchführung körperlichen Widerstand. Die Ärztin hatte zuvor ein Beruhigungsmittel verabreicht und ließ die Patientin festhalten, um die Injektion durchzuführen. Die Staatsanwaltschaft erhob Anklage wegen möglicher Verletzung der Sorgfaltskriterien – es war der erste Strafprozess dieser Art seit Einführung des niederländischen Sterbehilfegesetzes im Jahr 2002. Das Gericht sprach die Ärztin 2020 frei und bestätigte, dass sie im Einklang mit der vorausschauenden Verfügung und den gesetzlichen Vorgaben gehandelt habe. Der Fall löste eine breite ethische Debatte über die Grenzen und Auslegung solcher Verfügungen bei Demenz aus. In Folge dieses Urteils präzisierte der niederländische Oberste Gerichtshof, dass die Verabreichung eines Beruhigungsmittels vor Durchführung der Tötung auf Verlangen in bestimmten Fällen als Bestandteil ärztlicher Sorgfalt gelten kann, um mögliche Angst oder Abwehrreaktionen zu verhindern. Diese Rechtsprechung wurde anschließend in den „Euthanasia Code“ des Regional Euthanasia Review Committees (RTE) übernommen, der 2020 entsprechend angepasst wurde [18].

Seit Inkrafttreten des Gesetzes ist ein kontinuierlicher Anstieg der gemeldeten Fälle zu beobachten. Im Jahr 2024 registrierten die RTE 9958 Fälle aktiver Sterbehilfe, was einem Zuwachs von 10 % gegenüber dem Vorjahr entspricht. Der Anteil an der Gesamtsterblichkeit stieg von 5,4 % (2023) auf 5,8 % (2024; [19]). Die RTE gehen davon aus, dass dieser Trend mittelfristig anhalten wird. Zur Ursachenanalyse hat das Ministerium für Gesundheit, Wohlfahrt und Sport (*Ministerie van Volksgezondheid, Welzijn en Sport*, VWS) eine umfassende Untersuchung [20] in Auftrag gegeben, die in Kooperation des Radboud University Medical Center, des Universitair Medisch Centrum Utrecht und des Amsterdam UMC durchgeführt wird. Ziel ist die Ermittlung gesellschaftlicher, medizinischer und ethischer Faktoren, die zum Anstieg beitragen.

Die Fallverteilung im Jahr 2024 zeigt, dass 86,3 % der gemeldeten Fälle Patient:innen mit schweren somatischen Erkrankungen betrafen, insbesondere Krebs, neurologische Erkrankungen, Lungenerkrankungen und kardiovaskuläre Leiden. Daneben wurden 427 Fälle im Kontext von Demenzerkrankungen, 219 Fälle bei primär psychiatrisch bedingtem Leiden, 397 Fälle mit multiplen geriatrischen Syndromen sowie 232 Fälle in der Kategorie „sonstige Erkrankungen“ dokumentiert.

Das niederländische Modell umfasst zudem Sonderregelungen, die international Beachtung finden. So ist Sterbehilfe bei Jugendlichen ab 12 Jahren möglich, sofern die gesetzlichen Voraussetzungen erfüllt sind und eine elterliche Zustimmung vorliegt [21]. Gegenwärtig wird zudem intensiv über eine Ausweitung auf Personen mit sogenanntem vollendetem Leben diskutiert – also auf Menschen ohne medizinische Diagnose, die ihr Leben subjektiv als abgeschlossen betrachten. Ein weiteres ethisch relevantes Themenfeld bildet die Kombination von Sterbehilfe und Organspende. Unter bestimmten rechtlichen und medizinischen Voraussetzungen – darunter Durchführung in einem Krankenhaus und ausdrückliche Zustimmung – ist eine Organspende nach Sterbehilfe zulässig. Die Nederlandse Transplantatie Stichting (NTS; [22]) hat hierfür einen detaillierten Stufenplan [23] veröffentlicht, der sowohl die medizinischen Voraussetzungen als auch die ethischen Rahmenbedingungen und die Rollen der beteiligten Ärzt:innen, Ethikkommissionen und Transplantationskoordinator:innen definiert. Im Jahresbericht 2018 der RTE wurden 7 derartige Fälle dokumentiert.

Bemerkenswert ist, dass etwa 93 % aller niederländischen Sterbehilfefälle im häuslichen Umfeld und überwiegend durch Hausärzt:innen durchgeführt werden. Dies verdeutlicht sowohl die Integration der Sterbehilfe in die primärmedizinische Versorgung als auch die hohe gesellschaftliche Akzeptanz des Modells. Insgesamt manifestieren die Niederlande ein klar normiertes, medizinisch eingebettetes und gesellschaftlich breit reflektiertes Sterbehilfesystem, das in seiner Ausgestaltung innerhalb Europas singulär ist.

### Belgien

Obwohl Belgien sein Gesetzesmodell weitgehend an den niederländischen Regelungen orientierte, hat sich die Praxis dort in besonderer Weise ausgeweitet. Die 2014 erfolgte Aufhebung der Altersgrenze bei der aktiven Sterbehilfe sowie die zunehmende Anwendung bei psychischen Erkrankungen und nichtterminalen Leiden zeigen, dass Belgien heute zu den liberalsten Sterbehilferegimen weltweit zählt. Diese Entwicklung verdeutlicht exemplarisch die Tendenz zur gesellschaftlichen Normalisierung von Sterbehilfepraktiken, die in anderen Ländern – darunter auch Deutschland – mit besonderer Aufmerksamkeit verfolgt wird.

In Belgien ist die Tötung auf Verlangen seit Inkrafttreten des Gesetzes vom 23.09.2002 („Loi relative à l’euthanasie“) unter bestimmten, gesetzlich definierten Voraussetzungen zulässig. Das Gesetz gestattet Ärztinnen und Ärzten, auf ausdrückliches, freiwilliges und wiederholt geäußertes Verlangen einer urteilsfähigen Person deren Leben medizinisch zu beenden, sofern eine medizinisch aussichtslose Situation vorliegt und die betroffene Person unter körperlich oder psychisch unerträglichen, nicht linderbaren Beschwerden leidet. Die Durchführung unterliegt einem mehrstufigen Prüf- und Dokumentationsverfahren, das unter anderem die Konsultation eines zweiten, unabhängigen Arztes oder einer Ärztin sowie die schriftliche Festhaltung des Patientenverlangens umfasst.

Zum belgischen Rechtsrahmen gehört auch die Erweiterung auf Minderjährige. Der Wegfall einer Altersgrenze stellt somit eine Besonderheit dar. Seit einer Gesetzesänderung im Jahr 2014 ist Sterbehilfe auch für urteilsfähige Kinder und Jugendliche zulässig, sofern eine unheilbare körperliche Erkrankung und nicht behandelbare Schmerzen vorliegen. Die Zustimmung beider Elternteile sowie eine psychologische Begutachtung sind zwingende Voraussetzungen [24].

Die statistischen Daten der *Commission fédérale de contrôle et d’évaluation de l’euthanasie* zeigen seit 2002 einen kontinuierlichen Anstieg der gemeldeten Fälle. Im Jahr 2023 wurden 3423 Fälle registriert, was einem Zuwachs von 15 % im Vergleich zum Vorjahr entspricht. Damit lag der Anteil der Sterbehilfe an der Gesamtsterblichkeit bei rund 3,1 %. Die Mehrheit der Betroffenen war über 70 Jahre alt, rund 60 % litten an einer Krebserkrankung. Etwa 1,4 % der Fälle entfielen auf Demenzerkrankungen, ebenso 1,4 % auf psychiatrische Erkrankungen. In den beiden letztgenannten Kategorien ist ein signifikanter Anstieg zu beobachten: Die Zahl der dokumentierten Fälle bei Demenzpatient:innen stieg von 162 (2019) auf 328 (2023), bei Menschen mit psychischen Erkrankungen von 68 auf 138 im gleichen Zeitraum.

Das belgische Recht erlaubt zudem die Abgabe einer vorausschauenden Verfügung zur Sterbehilfe. Diese ist rechtlich bindend, sofern sie von einer volljährigen oder für mündig erklärten Person in urteilsfähigem Zustand freiwillig, wohlüberlegt und wiederholt verfasst wurde. Sie muss schriftlich fixiert und eindeutig formuliert sein sowie festlegen, dass Sterbehilfe gewährt werden soll, wenn eine medizinisch aussichtslose Situation mit schwerem, unheilbarem Leiden und irreversiblem Bewusstseinsverlust vorliegt [25]. Minderjährigen ist die Abgabe einer solchen Vorausverfügung nicht gestattet; bei ihnen ist ausschließlich eine aktuelle, unter strengen Bedingungen geäußerte Bitte zulässig.

Im September 2024 erhielt die Debatte um eine Ausweitung des Gesetzes neue Impulse, als Abgeordnete der liberalen Partei *Open VLD* einen Gesetzesentwurf (DOC 56 0183/001) einbrachten, der die Anwendung der Sterbehilfe explizit auf Personen mit fortgeschrittener Demenz ausdehnen soll, sofern eine entsprechende Patientenverfügung vorliegt. Die Befürworter argumentieren, dass viele Betroffene sich aus Sorge vor einem Verlust der Einwilligungsfähigkeit zu früh für Sterbehilfe entscheiden und so möglicherweise Lebensqualität einbüßen. Kritiker, darunter das *Institut Européen de Bioéthique* (EIB; [26]), warnen hingegen vor einer Verschiebung von Selbst- zu Fremdbestimmung, insbesondere bei vulnerablen Gruppen. Sie verweisen auf Risiken, wenn ökonomische Faktoren [27] wie hohe Behandlungskosten im Alter die Entscheidung beeinflussen könnten. Die Internetseite der „Ageing Equal“ publizierte einen Beitrag, wonach rund 40 % der Belgier Einsparungen „durch das Nicht-Verabreichen kostenintensiver Behandlungen, die das Leben von Über-85-Jährigen verlängern“, befürworteten [28].

### Kanada

In Kanada wurde die gesetzliche Grundlage für die medizinisch assistierte Sterbehilfe (*Medical Assistance in Dying*, MAID; [29]) mit dem Inkrafttreten des Gesetzes *Bill C‑14* [30] im Juni 2016 geschaffen. Das Gesetz reagierte auf das Urteil *Carter v. Canada* des Obersten Gerichtshofs, in dem das bis dahin geltende strafrechtliche Verbot der aktiven Sterbehilfe für verfassungswidrig erklärt wurde. *Bill C‑14* legalisierte sowohl den ärztlich assistierten Suizid als auch die Tötung auf Verlangen (*Voluntary Euthanasia*) für volljährige, einwilligungsfähige Personen, deren Tod als „vernünftigerweise vorhersehbar“ eingestuft wurde und die von einem gravierenden, unheilbaren Leiden betroffen waren.

Mit *Bill C‑7 *[31], verabschiedet im März 2021, wurde der Zugang zu MAID substanziell erweitert. Die Voraussetzung eines absehbaren Todes entfiel, sodass auch Personen ohne terminale Diagnose, jedoch mit chronisch schweren Erkrankungen Anspruch auf MAID erhielten. Für diese neu einbezogene Gruppe (sog. *Track 2*) wurde eine obligatorische Bedenkfrist von 90 Tagen eingeführt. Parallel entfiel für terminal erkrankte Personen (*Track 1*) die zuvor geltende 10-tägige Wartefrist, sodass eine Durchführung am Tag der Antragstellung möglich wurde.

Die amtlichen Statistiken zeigen eine kontinuierliche Zunahme der MAID-Fälle. Im Jahr 2023 [32] wurden 19.660 Anträge verzeichnet, von denen 15.343 zur MAID führten – ein Anstieg um 15,8 % gegenüber dem Vorjahr. 95,9 % der durchgeführten Verfahren entfielen auf *Track 1*, lediglich 4,1 % auf *Track 2*. In über 99 % der Fälle erfolgte die MAID durch intravenöse Verabreichung eines tödlichen Medikaments durch medizinisches Fachpersonal, womit die Tötung auf Verlangen faktisch die dominante Form darstellt [33]. Der assistierte Suizid, bei dem die Einnahme eigenständig erfolgt, wird nur in Ausnahmefällen gewählt (< 1 %). 94,5 % der Verfahren wurden von Ärzt:innen vollzogen, 5,5 % von qualifizierten Pflegefachpersonen (Nurse Practitioners). Auffällig ist die Konzentration der Durchführung auf einen kleinen Kreis: 89 Fachpersonen verantworteten mehr als ein Drittel aller MAID-Fälle im Jahr 2023.

Zunehmend wird MAID auch als Reaktion auf psychosoziale Belastungslagen beantragt. Laut offiziellen Berichten gaben 2022 rund 17,1 % der Betroffenen Einsamkeit und soziale Isolation als Hauptmotiv an [32]. Weitere häufig genannte Gründe waren das Empfinden, eine Belastung für Angehörige darzustellen (34 %) sowie der Verlust der Fähigkeit zu sinnstiftenden Aktivitäten (82,1 %). Einzelne dokumentierte Fälle betrafen Personen, die unter Obdachlosigkeit, Überschuldung oder fehlender sozialer Unterstützung litten. Diese Entwicklungen werfen ethische Fragen hinsichtlich des Schutzes vulnerabler Gruppen sowie der Gefahr einer gesellschaftlichen Normalisierung von Sterbehilfe als Lösung nichtmedizinischer Problemlagen auf [29, 34].

Auch gesundheitsökonomische Aspekte finden Eingang in die Debatte. Eine Kostenanalyse des *Parliamentary Budget Office* („Cost Estimate for Bill C‑7“, 20.10.2020) prognostizierte Einsparungen im Gesundheitssystem in Höhe von 89,6 Mio. CAD durch die bestehende Gesetzeslage sowie bis zu 149 Mio. CAD bei Umsetzung der erweiterten Regelungen [35]. Zwar betonten die Autor:innen, dass die Analyse nicht als Argumentation für eine Kostenreduktion durch Sterbehilfe verstanden werden solle, dennoch hielten sie fest, dass die Ausweitung des Zugangs zu MAID zu einer Nettoverringerung der Gesundheitsausgaben auf Provinzebene führe.

Die Entwicklung in Kanada verläuft im internationalen Vergleich besonders dynamisch. Innerhalb weniger Jahre nach der Legalisierung hat sich MAID von einer Ausnahme zu einer breit akzeptierten Praxis entwickelt, deren Anwendungsbereich stetig erweitert wird. Bemerkenswert ist dabei, dass in Kanada nahezu ausschließlich die aktive Form der Sterbehilfe praktiziert wird: Über 99 % der Verfahren erfolgen durch die Verabreichung eines tödlichen Medikaments durch Ärzt:innen oder qualifizierte Pflegefachpersonen. Der ärztlich assistierte Suizid im engeren Sinne spielt dagegen kaum eine Rolle.

## Aktuelle Entwicklung in Frankreich sowie England und Wales

Mit dem *Loi Claeys-Leonetti* von 2016 führte Frankreich erstmals eine gesetzliche Grundlage für die „tiefe und kontinuierliche Sedierung bis zum Tod“ ein. Diese Maßnahme ist auf ausdrücklichen Wunsch unheilbar erkrankter Patient:innen zulässig, deren Lebensende absehbar ist, und dient ausschließlich der Leidenslinderung ohne aktive Lebensverkürzung [36].

Im Mai 2025 verabschiedete die Assemblée Nationale eine Reform, die den Anwendungsbereich über die Sedierung hinaus auf die kontrollierte Abgabe tödlich wirkender Substanzen ausdehnt. Diese dürfen entweder eigenständig durch die sterbewillige Person eingenommen oder – bei körperlicher Unfähigkeit – von medizinischem Personal verabreicht werden. Die gesetzlichen Zugangsvoraussetzungen sind: Volljährigkeit, französische Staatsangehörigkeit oder dauerhafter Aufenthalt, ein freier und informierter Wille sowie das Vorliegen einer unheilbaren, fortgeschrittenen Erkrankung mit dauerhaftem, unerträglichem Leiden. Psychische Leiden allein begründen keinen Anspruch. Zum Vergleich sei angemerkt, dass in den Vereinigten Staaten ausschließlich der ärztlich assistierte Suizid in einzelnen Bundesstaaten zulässig ist, während die Tötung auf Verlangen verboten bleibt; aufgrund der vergleichsweise geringen Fallzahlen und der rein assistierten Ausgestaltung wird dieses Modell hier im Länderteil nicht gesondert dargestellt.

Das Verfahren sieht die Einschaltung eines *Collège de Concertation* vor, bestehend aus 2 Ärzt:innen und einer Pflegefachkraft, sowie eine dokumentierte Entscheidungsfindung [37]. Eine Besonderheit der französischen Regelung ist die Einführung eines Straftatbestandes, der die gezielte Erschwerung des Zugangs zu Suizidhilfe unter Strafe stellt (bis zu 2 Jahre Freiheitsstrafe).

In England und Wales ist die Beihilfe zum Suizid gemäß dem *Suicide Act 1961* strafbar und kann mit Freiheitsstrafen von bis zu 14 Jahren geahndet werden. Im Oktober 2024 wurde mit der *Terminally Ill Adults (End of Life) Bill* [38] ein Gesetzentwurf in das parlamentarische Verfahren eingebracht, der eine begrenzte Zulassung der Suizidhilfe vorsieht [39]. Anspruch hätten volljährige Personen mit Wohnsitz in England oder Wales seit mindestens einem Jahr, die an einer unheilbaren Erkrankung leiden und voraussichtlich innerhalb von 6 Monaten versterben werden. Das Vorliegen schwerer Schmerzen wäre nicht erforderlich; eine selbst herbeigeführte Erkrankung – etwa durch absichtliche Nahrungsverweigerung – würde die Antragstellung ausschließen.

Das vorgesehene Verfahren ist 3‑stufig:

1. Untersuchung und Aufklärung durch einen *Coordinating Doctor,*
2. unabhängige Zweitbegutachtung durch einen *Independent Doctor,*
3. Prüfung des Antrags durch ein *Assisted Dying Review Panel*, dem ein staatlich bestellter Kommissar die Unterlagen vorlegt.

Nach einer obligatorischen 14-tägigen Bedenkfrist könnte die Einnahme eines tödlichen Mittels unter ärztlicher Aufsicht erfolgen. Detailfragen – etwa die Unterstützung bei physischer Unfähigkeit oder der Umgang mit psychischen Erkrankungen – sollen in einem ergänzenden *Code of Practice* geregelt werden.

## Diskussion

### Vergleich der analysierten Länder

Die vergleichende Betrachtung der internationalen Regelungen zeigt deutliche Unterschiede im rechtlichen und ethischen Umgang mit Sterbehilfe und Suizidassistenz. Während die Niederlande, Belgien und Kanada in den vergangenen Jahren eine schrittweise Ausweitung der Zulassungskriterien und Anwendungsbereiche verzeichneten, verfolgen England und Wales restriktive Modelle. Die Schweiz nimmt eine Sonderrolle ein, da dort die Suizidassistenz seit Jahrzehnten auf Grundlage eines Strafrechtsartikels praktiziert wird, ohne dass eine spezifische gesetzliche Regulierung besteht. Diese unterschiedlichen Modelle spiegeln verschiedene gesellschaftliche Prioritäten wider – zwischen dem Schutz vulnerabler Gruppen, der Sicherung individueller Autonomie und der Vermeidung von Missbrauchsrisiken. Neben den rechtlichen Regelungen unterscheiden sich auch die praktischen Formen der Durchführung deutlich zwischen den Ländern.

In Belgien und Kanada erfolgt die Sterbehilfe überwiegend durch Ärzt:innen, teils unterstützt durch spezialisierte Pflegefachpersonen, meist im häuslichen oder palliativmedizinischen Umfeld.

Ein Vergleich mit den Vereinigten Staaten verdeutlicht die Besonderheiten des kanadischen Modells: Während in Kanada nahezu ausschließlich Tötung auf Verlangen praktiziert wird, ist in den USA ausschließlich der ärztlich assistierte Suizid erlaubt. Die Fallzahlen bleiben dort deutlich niedriger. In den Vereinigten Staaten ist ein begrifflicher Wandel zu beobachten: Befürworter vermeiden zunehmend die Bezeichnung „assisted suicide“ und sprechen stattdessen von „medical aid in dying“, um die Praxis sprachlich zu entstigmatisieren [40]. In Kanada hingegen bezeichnet MAID ein gesetzlich definiertes Regelungskonzept und ist nicht primär Ausdruck sprachpolitischer Strategie. In der Schweiz sind Ärzt:innen ebenfalls in die Suizidassistenz eingebunden, wobei die Durchführung häufig durch Sterbehilfeorganisationen koordiniert wird. Diese Unterschiede in der organisatorischen Einbindung zeigen, wie stark nationale Gesundheitssysteme und professionelle Rollenbilder die konkrete Praxis der Sterbehilfe prägen. Ein Vergleich der bestehenden internationalen Modelle verdeutlicht, dass jedes System spezifische Stärken und Schwächen aufweist.

**Abb. 1:**
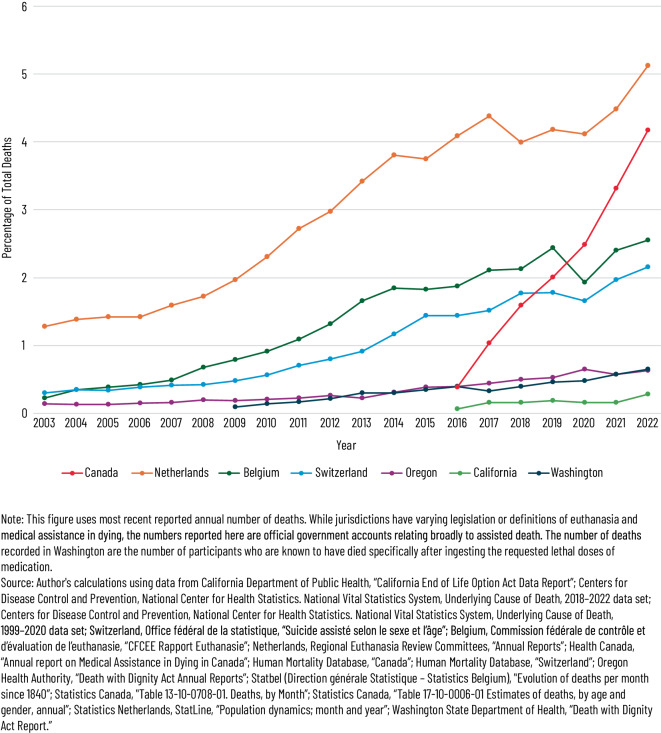
Anteil assistierter Todesfälle an der Gesamtsterblichkeit nach Ländern (2003–2022). Abbildung mit freundlicher Genehmigung verwendet aus [41], publiziert unter der Lizenz CC BY-NC-ND 4.0 [46]. Verwendung offizieller Daten aus Health Canada, Statistics Netherlands, Commission fédérale de contrôle et d’évaluation de l’euthanasie (Belgien), Office fédéral de la statistique (Schweiz) und Centers for Disease Control and Prevention (USA). *Hinweis:* Die US-Bundesstaaten Oregon, Washington und Kalifornien werden im Beitrag nicht ausführlich dargestellt. Ihre Aufnahme in der Abbildung erfolgt aus Gründen der quantitativen Vergleichbarkeit und basiert auf der Originalquelle

Wie Abb. 1 (übernommen aus [41]) zeigt, ist in den dort dargestellten Ländern, in denen Sterbehilfe bzw. assistierter Suizid gesetzlich geregelt oder zulässig ist, über die Zeit überwiegend ein kontinuierlicher Anstieg des Anteils assistierter Todesfälle an allen Todesfällen zu beobachten. Besonders deutlich fällt die Zunahme in Kanada und den Niederlanden aus, während die Schweiz und Belgien einen stabileren Verlauf zeigen. In den US-Bundesstaaten Oregon und Washington bleibt der Anteil trotz langjähriger Zulassung vergleichsweise niedrig. Diese Entwicklungen spiegeln nicht nur unterschiedliche gesetzliche Rahmenbedingungen wider, sondern auch kulturelle und medizinische Unterschiede in der praktischen Umsetzung.

In Ländern wie den Niederlanden, Belgien oder Kanada ermöglichen klar definierte gesetzliche Verfahren und Meldepflichten eine hohe Transparenz und rechtliche Sicherheit. Diese Strukturen stärken das Vertrauen der Bevölkerung und schaffen nachvollziehbare Kontrollmechanismen, die Missbrauch vorbeugen sollen. Zugleich wird jedoch diskutiert, dass eine zunehmende Liberalisierung, etwa durch erweiterte Zugangskriterien oder die Einbeziehung nichtterminaler Erkrankungen, zu einer schleichenden Normalisierung der Sterbehilfe führen kann. Restriktivere Systeme – wie in England und Wales – setzen stärker auf den Schutz vulnerabler Gruppen, können jedoch unbeabsichtigt dazu beitragen, dass schwerkranke oder leidende Personen ohne realistische Handlungsoptionen bleiben und dadurch in Grauzonen gedrängt werden. Damit zeigt sich, dass die Herausforderung nicht in der Wahl zwischen restriktivem oder liberalem Ansatz liegt, sondern in der Balance von Selbstbestimmung, Schutz und gesellschaftlicher Verantwortung.

### Die Debatte in Deutschland im Vergleich

Die rechtliche Regelung der Sterbehilfe in Deutschland bewegt sich im Spannungsfeld zwischen dem verfassungsrechtlich garantierten Selbstbestimmungsrecht und strafrechtlichen Restriktionen. Im internationalen Vergleich zeigen sich 2 grundsätzlich unterschiedliche Entwicklungsrichtungen: Während Länder wie die Niederlande, Belgien oder Kanada den Zugang zu Sterbehilfe und Suizidassistenz schrittweise erweitert und durch verbindliche Prüf- und Dokumentationsverfahren abgesichert haben, verfolgen andere Länder – etwa England, Irland oder Österreich – weiterhin restriktive Ansätze, die den Schutz vulnerabler Gruppen betonen. Diese Gegenüberstellung verdeutlicht, dass eine gesetzliche Liberalisierung nicht automatisch verantwortliche Praxis und Schutz vor Fehlentwicklungen gewährleistet, ebenso wenig wie ein rein restriktiver Ansatz per se Vulnerabilitäten vermeidet. Entscheidend ist, in welchem Maß rechtliche, medizinische und gesellschaftliche Kontrollmechanismen ineinandergreifen, um sowohl individuelle Autonomie als auch Fürsorge und Schutzpflichten zu sichern.

Für Deutschland ergibt sich daraus die Aufgabe, das vom Bundesverfassungsgericht bestätigte Recht auf selbstbestimmtes Sterben mit klaren prozeduralen Standards zu verbinden, die Transparenz schaffen, Missbrauch verhindern und die Suizidassistenz in ein verantwortbares medizinisch-ethisches Gesamtkonzept einbetten. Im Unterschied zu den international vergleichend dargestellten Ländern befindet sich Deutschland nach wie vor in einem Übergangszustand: Zwar hat das Bundesverfassungsgericht ein Grundrecht auf selbstbestimmtes Sterben anerkannt, doch fehlt bislang eine gesetzliche Regelung, die die praktische Umsetzung verbindlich ausgestaltet. Die Perspektive, ein Suizid sei Ausdruck höchstpersönlicher Freiheit, blendet aus, dass Freiheit immer auch Verantwortung beinhaltet – sowohl für die betroffene Person selbst als auch für ihre sozialen Beziehungen und das medizinische Umfeld [42]. In vielen Debatten wird der Autonomiebegriff dabei auf individuelle Entscheidungsfreiheit verengt, ohne die existenziellen Lebensbezüge mitzudenken, in denen Menschen eingebettet sind – etwa Abhängigkeiten, Bindungen, Fürsorge- und Beziehungskontexte [43].

Zudem wird häufig übersehen, dass die aktive Beteiligung Dritter an einer Tötungshandlung ein grundlegendes ethisches und anthropologisches Problem darstellt, weil sie professionelle und persönliche Rollen berührt und das gesellschaftliche Verständnis von Mitmenschlichkeit, Fürsorge und medizinischer Verantwortung herausfordert [44]. Ungeachtet dessen bleibt die aktive Tötung auf Verlangen nach § 216 StGB strafbar. Der Bundesgerichtshof hat 2022 (BGHSt 67, 95; [42]) allerdings angedeutet, dass § 216 StGB bei verfassungskonformer Auslegung nicht anzuwenden sei, wenn eine freiverantwortliche Suizidentscheidung faktisch nicht mehr eigenständig umgesetzt werden kann.

Mehrere fraktionsübergreifende Gesetzesentwürfe im Bundestag, die von einem weitgehenden Verbot der geschäftsmäßigen Suizidhilfe bis hin zu einer kontrollierten Abgabe tödlich wirkender Substanzen reichten (BT-Drs. 20/904, 20/2293, 20/2332), scheiterten bislang. In der Praxis ist die geschäftsmäßige Suizidhilfe unter bestimmten Voraussetzungen nicht strafbar, wird jedoch durch arzneimittel- und betäubungsmittelrechtliche Vorschriften erheblich erschwert.

Vor diesem Hintergrund konzentriert sich die deutsche Debatte auf die assistierte Selbsttötung, bei der die tödliche Substanz von der sterbewilligen Person selbst eingenommen wird. Eine aktive Tötung durch Dritte – selbst auf ausdrückliches Verlangen – bleibt strafbar und wird mehrheitlich als mit dem Schutz des Lebens unvereinbar angesehen.

Diskutiert wird eine gesetzliche Neuregelung, die Kriterien aus anderen europäischen Modellen kombiniert – etwa anhaltend starke Schmerzen (Frankreich) oder eine Lebenserwartung unter 6 Monaten (England) als alternative Zugangsvoraussetzungen. Ein geregeltes, dokumentiertes Verfahren ist deshalb unabdingbar, weil es Transparenz, Nachvollziehbarkeit und Rechtssicherheit gewährleistet – sowohl für die sterbewilligen Personen als auch für die beteiligten Fachkräfte. Internationale Erfahrungen zeigen, dass mehrstufige Prüfungen, verpflichtende Meldungen und eine unabhängige Nachprüfung zentrale Schutzmechanismen darstellen: In den Niederlanden prüfen die Regionalen Prüfungskommissionen (RTE; [18]) jeden gemeldeten Fall anhand gesetzlich definierter Sorgfaltskriterien; in Belgien erfolgt die Kontrolle durch die Föderale Kontroll- und Bewertungskommission [43] und in Kanada sichern gesetzliche Meldepflichten sowie standardisierte Assessments Transparenz und Nachvollziehbarkeit ([32]; RTE Annual Report 2024 [18]; Föderale Kontroll- und Bewertungskommission Sterbehilfe | Volksgezondheid [43]). Durch klar definierte Verantwortlichkeiten und eine interdisziplinäre Begutachtung lassen sich zudem Fehlentscheidungen und ungleiche Anwendungspraxen vermeiden. Damit bilden verbindliche Standards einen zentralen Bestandteil jeder ethisch und rechtlich tragfähigen Regelung von Sterbehilfe und Suizidassistenz.

Besonders komplex ist der Umgang mit Suizidwünschen bei Menschen mit psychischen Erkrankungen. In diesen Fällen stellen sich besondere Herausforderungen hinsichtlich der Beurteilung der Freiverantwortlichkeit, der Abgrenzung zwischen therapierbarer Suizidalität und einem anhaltenden Sterbewunsch sowie der ethischen Verantwortung der behandelnden Fachkräfte. Ähnliche ethische und rechtliche Fragen ergeben sich im Zusammenhang mit Sterbewünschen von Menschen mit Demenz. In einigen Ländern, etwa in den Niederlanden oder Belgien, sind Vorausverfügungen unter bestimmten Voraussetzungen zulässig, während andere Staaten die Thematik bislang nicht ausdrücklich gesetzlich geregelt haben. Der Umgang mit demenziell erkrankten Personen verdeutlicht in besonderer Weise die Spannungsfelder zwischen Autonomie, Fürsorge und Schutzpflicht.

Die Deutsche Gesellschaft für Psychiatrie und Psychotherapie, Psychosomatik und Nervenheilkunde (DGPPN) fordert in ihrer Stellungnahme von Juli 2024 [44] eine gesetzliche Regelung, die psychiatrische Expertise systematisch in die Prüfung der Freiverantwortlichkeit einbindet. Sie warnt vor einer vorschnellen Liberalisierung ohne flankierende Schutz- und Präventionsmaßnahmen und plädiert für eine interdisziplinäre Begutachtung sowie eine klare Rollenabgrenzung zwischen Suizidassistenz und Suizidprävention.

Eine fundierte gesellschaftliche und politische Diskussion zur Suizidassistenz erfordert eine verlässliche Datengrundlage. Während Länder wie die Niederlande und Kanada seit Jahren detaillierte Register führen und regelmäßig öffentlich zugängliche Berichte zur Praxis der Sterbehilfe veröffentlichen, fehlt in Deutschland bislang eine separate statistische Erfassung von Suizidassistenzfällen. Derzeit werden diese gemeinsam mit „regulären“ Suiziden in der ICD-10-Kategorie der Todesursachenstatistik geführt, was eine differenzierte Analyse verhindert.

Aktuell fördert das Bundesministerium für Gesundheit das Projekt RegAS [45], das ein bundesweites Register für Suizidassistenzfälle auf freiwilliger Basis aufbauen soll. Dieses Vorhaben stellt einen ersten wichtigen Schritt dar und könnte langfristig als Modell für eine offizielle, verpflichtende Erfassung dienen. Eine zentrale und kontinuierliche Dokumentation wäre nicht nur aus Gründen der Transparenz geboten, sondern auch Voraussetzung, um Entwicklungen, Indikationen und Motivlagen besser zu verstehen und Fehlentwicklungen frühzeitig zu erkennen.

### Verhältnis von Selbstbestimmung, Fürsorge und gesellschaftlicher Verantwortung

Der internationale Vergleich verdeutlicht, dass sich die rechtlichen und gesellschaftlichen Grenzen der Sterbehilfe in den vergangenen Jahren erheblich verschoben haben. Die zunehmende Liberalisierung in vielen Ländern zeigt, wie rasch sich eine ursprünglich eng gefasste Ausnahme zu einer etablierten Praxis entwickeln kann. Diese Entwicklung wirft grundlegende Fragen nach dem Verhältnis von Selbstbestimmung, Fürsorge und gesellschaftlicher Verantwortung auf.

Eine offene Gesellschaft steht in der Verantwortung, Menschen am Lebensende oder mit schweren chronischen Erkrankungen nicht allein zu lassen. Klinische Erfahrungen zeigen, dass eine gut zugängliche palliative Betreuung – mit suffizienter Symptomkontrolle, psychosozialer Begleitung, Beziehungspflege und gelebter Fürsorge – dazu beitragen kann, dass Sterbewünsche sich verändern und oft an Dringlichkeit verlieren. Die Haltung der Palliativmedizin spielt auch in der internationalen Diskussion eine zentrale Rolle. In den meisten Ländern versteht sich die Palliativmedizin als eigenständige Disziplin mit dem Ziel, Leid zu lindern und Lebensqualität bis zuletzt zu erhalten – nicht aber aktiv den Tod herbeizuführen. Dennoch wird zunehmend diskutiert, wie sich die Palliativversorgung und die Sterbehilfe zueinander verhalten. In einigen Staaten, etwa Kanada oder Belgien, sind Ärzt:innen mit palliativmedizinischem Hintergrund teilweise auch in Verfahren der Sterbehilfe eingebunden, während in anderen Ländern eine klare Trennung zwischen palliativmedizinischer Betreuung und Suizidassistenz besteht. Diese unterschiedlichen Modelle zeigen, dass sich die Rolle der Palliativmedizin im Spannungsfeld zwischen Fürsorge, Autonomie und ärztlicher Ethik weiterhin im Wandel befindet.

Eine starke Palliativ- und Hospizversorgung, ergänzt durch psychologische und soziale Unterstützungsangebote sowie eine Kultur des Miteinanders, kann Menschen in belastenden Lebens- und Krankheitssituationen stabilisieren und ihnen ermöglichen, ihre Wünsche, Prioritäten und Entscheidungen in Ruhe zu reflektieren. Der Fokus liegt dabei auf einer Begleitung, die Menschen am Lebensende unterstützt, ihr Leben und Sterben als möglichst getragen und verbunden zu erleben – unabhängig davon, ob sie sich letztlich für oder gegen Sterbehilfe entscheiden. Suizidassistenz kann Ausdruck eines individuellen, wohlüberlegten Entschlusses sein – etwa im vertrauensvollen Rahmen zwischen Patient:innen und ihren behandelnden Ärzt:innen.

Internationale Entwicklungen zeigen, dass eine schrittweise Ausweitung von Sterbehilferegelungen zu Formen gesellschaftlicher Normalisierung führen kann. Damit verbunden ist die Sorge, dass mit der Etablierung routinierter oder kommerzieller Strukturen Erwartungen entstehen könnten, die insbesondere vulnerable Gruppen zusätzlich unter Druck setzen. Die Erfahrungen anderer Länder machen deutlich, wie schmal der Grat zwischen mitmenschlicher Unterstützung und der Institutionalisierung des Todes sein kann. Eine ethisch verantwortete Sterbehilfepolitik kann daher nicht allein auf gesetzliche Regelungen setzen, sondern muss zugleich auf die Stärkung von Fürsorge, Beziehung und Solidarität bauen – und auf eine Gesellschaft, die das Leben bis zuletzt begleitet. Zugleich zeigen die Erfahrungen aus den Niederlanden, Belgien und Kanada, dass transparente Prüfverfahren und verbindliche Dokumentationspflichten maßgeblich zur Missbrauchsvermeidung und zur öffentlichen Akzeptanz beitragen können.

Für Deutschland ergibt sich daraus die Aufgabe, das vom Bundesverfassungsgericht (BVerfGE 153, 182) anerkannte Recht auf selbstbestimmtes Sterben in ein konsistentes Schutzkonzept zu integrieren. Eine künftige gesetzliche Regelung sollte klare prozedurale Vorgaben schaffen, die die Freiverantwortlichkeit absichern, Missbrauch verhindern und zugleich den Schutz vulnerabler Gruppen gewährleisten. Dabei können internationale Modelle als Orientierungsrahmen dienen, etwa hinsichtlich klar definierter Zugangsvoraussetzungen, ärztlicher Zweitbegutachtungen und verpflichtender Meldesysteme.

Hinweise auf politische Aktivitäten, darunter die im Bundestag wiederaufgenommene Gesetzesinitiative und das vom Bundesministerium für Gesundheit geförderte Projekt RegAS [45] zur Einrichtung eines Registers für Suizidassistenzfälle, zeigen, dass der Gesetzgebungsprozess fortbesteht. Eine zukünftige Regelung sollte die Wertschätzung des Lebens und das Recht auf selbstbestimmtes Sterben in ein ausgewogenes Verhältnis setzen. Die Möglichkeit der Suizidassistenz darf nicht zur Antwort auf gesellschaftliche, medizinische oder soziale Defizite werden. Eine rechtssichere, ethisch reflektierte und praktisch anwendbare Grundlage muss Autonomie, Fürsorge und Schutzpflichten gleichermaßen sichern – und damit die Balance zwischen Freiheit und Verantwortung wahren.

## References

[1] Strafgesetzbuch (StGB), in der Fassung der Bekanntmachung vom 13. November 1998 (BGBl. I S. 3322), zuletzt geändert durch Artikel 1 des Gesetzes vom 26. Juli 2023 (BGBl. 2023 I Nr. 203).

[2] Ethikrat N (2006) Selbstbestimmung und Fürsorge am Lebensende. https://www.ethikrat.org/fileadmin/Publikationen/Stellungnahmen/Archiv/Stellungnahme_Selbstbestimmung_und_Fuersorge_am_Lebensende.pdf. Accessed 23.10.2025

[3] Gronemeyer R (2011) Gastbeitrag: Das Lebensende wird zur Planungsaufgabe. https://www.tagesspiegel.de/wissen/das-lebensende-wird-zur-planungsaufgabe-1916614.html

[4] Bundesverfassungsgericht (2020) Urteil vom 26. Februar 2020. https://www.bundesverfassungsgericht.de/SharedDocs/Entscheidungen/DE/2020/02/rs20200226_2bvr234715.html. Accessed 23.10.2025

[5] Taupitz J (2020) Das Recht auf selbstbestimmtes Sterben – Kommentar zum Urteil des Bundesverfassungsgerichts vom 26. Februar 2020. Medizinrecht 38:401–406

[6] Gronemeyer R (2007) Sterben in Deutschland: Wie wir dem Tod wieder einen Platz in unserem Leben einräumen können. S. Fischer

[7] Mroz S, Dierickx S, Deliens L, Cohen J, Chambaere K (2021) Assisted dying around the world: a status quaestionis. Ann Palliat Med 10:3540–3553. 10.21037/apm-20-637 32921084 10.21037/apm-20-637

[8] Cohen J, Van Landeghem P, Carpentier N, Deliens L (2014) Public acceptance of euthanasia in Europe: a survey study in 47 countries. Int J Public Health 59:143–156. 10.1007/s00038-013-0461-6 23558505 10.1007/s00038-013-0461-6

[9] Statistik Bf (2024) Todesursachenstatistik 2023 - GNP Veröffentlichungen. https://www.bfs.admin.ch/news/de/2024-0101. Accessed 23.10.2025

[10] Gesundheit Bf (2021) Faktenblatt - Demographische Entwicklung und Pflegebedarf. https://swissvotes.ch/attachments/45cca5a460712850f5ab21abacde88f8a686f26e2cf97c0647999896d756dc0f. Accessed 23.10.2025

[11] Resort TL (2025) The Last Resort. https://www.thelastresort.ch/deutsch/. Accessed 23.10.2025

[12] Fernsehen SSRu (2025) Erster Einsatz der Todeskapsel Sarco – die wichtigsten Antworten. https://www.srf.ch/news/schweiz/mehrere-strafverfahren-laufen-erster-einsatz-der-todeskapsel-sarco-die-wichtigsten-antworten. Accessed 23.10.2025

[13] Uwe Güth ARS, Edouard Battegay (2024) Ist Suizidhilfe in Vereinshand Teil des eidgenössischen Brauchtums? ARS MEDICI, https://www.rosenfluh.ch/media/arsmedici/2024/24/Die-Zukunft-der-Suizidhilfe-in-der-Schweiz-Teil-3-Ist-Suizidhilfe-in-Vereinshand-Teil-des-eidgenoessischen-Brauchtums.pdf:604–607.

[14] Zürich SK (2025) Regierungsrat befürwortet die Sterbehilfe in allen Alters- und Pflegeheimen – lehnt die zu weit gehende Volksinitiative aber ab. https://www.zh.ch/de/news-uebersicht/medienmitteilungen/2025/02/regierungsrat-befuerwortet-die-sterbehilfe-in-allen-alters-und-pfelgeheimen-lehnt-die-zu-weit-gehende-volksinitiative-aber-ab.html. Accessed 23.10.2025

[15] EXIT (2025) Recht auf Selbstbestimmung am Lebensende auch im Altersheim. https://selbstbestimmung-auch-im-heim.ch/initiative. Accessed 23.10.2025

[16] Netherlands Go Euthanasia. In: https://www.government.nl/topics/euthanasia. Zugegriffen: 23.10.2025

[17] (RTE) RKfS In: https://www.euthanasiecommissie.nl/. Zugegriffen: 23.10.2025

[18] (RTE) RKfS Annual Reports. In: https://english.euthanasiecommissie.nl/the-committees/annual-reports. Zugegriffen: 23.10.2025

[19] (RTE) RKfS (2024) Jahresbericht 2024. https://english.euthanasiecommissie.nl/documents/annual-reports/2002/annual-reports/annual-reports. Accessed 23.10.2025

[20] Radboudumc (2025) Radboudumc leads study into developments in euthanasia practice. https://www.radboudumc.nl/en/news-items/2025/radboudumc-leads-study-into-developments-in-euthanasia-practice

[21] Ärzteblatt RD (2024) Niederlande erlauben ab Februar Sterbehilfe auch für Kinder – Deutsches Ärzteblatt. https://www.aerzteblatt.de/news/niederlande-erlauben-ab-februar-sterbehilfe-auch-fuer-kinder-2ab5e31a-c7fd-411e-ab77-110aa4f5f715

[22] Stichting NT Organspendeverfahren. In: https://transplantatiestichting.nl/donatieprocedure/organen?lang=de. Zugegriffen: 23.10.2025

[23] Stichting NT (2025) Informatie voor professionals over orgaandonatie bij euthanasie. https://transplantatiestichting.nl/kennisdossiers/euthanasie

[24] Ärzteblatt RD (2014) Aktive Sterbehilfe in Belgien: Ausweitung auf Minderjährige beschlossen – Deutsches Ärzteblatt. Deutsches Ärzteblatt. https://www.aerzteblatt.de/archiv/aktive-sterbehilfe-in-belgien-ausweitung-auf-minderjaehrige-beschlossen-9caba079-fc48-47ad-832b-4c0895fca4de

[25] Publique S (2016) Sterbehilfe. https://www.health.belgium.be/de/gesundheit/sorgen-sie-fuer-sich-selbst/lebensanfang-und-lebensende/sterbehilfe

[26] Bioéthique IEd (2025) Proposed extension of Belgian euthanasia law: critical feedback from the Netherlands - European Institute of Bioethics. https://www.ieb-eib.org/en/news/end-of-life/euthanasia-and-assisted-suicide/proposed-extension-of-belgian-euthanasia-law-critical-feedback-from-the-netherlands-2329.html. Accessed 23.10.2025

[27] Bioéthique IEd (2025) L’euthanasie au Canada, réponse à la vulnérabilité sociale et économique des citoyens ? - Institut Européen de Bioéthique. https://www.ieb-eib.org/fr/actualite/fin-de-vie/euthanasie-et-suicide-assiste/l-euthanasie-au-canada-reponse-a-la-vulnerabilite-sociale-et-economique-des-citoyens-1844.html. Accessed 23.10.2025

[28] Europe AP (2019) 40 % of Belgians in favour of stopping care after 85 years old. https://ageing-equal.org/40-of-belgians-in-favour-of-stopping-care-after-85-years-old/. Accessed 23.10.2025

[29] Konder RM, Christie T (2019) Medical Assistance in Dying (MAiD) in Canada: A Critical Analysis of the Exclusion of Vulnerable Populations. Healthc Policy 15:28–38. 10.12927/hcpol.2019.26073 32077843 10.12927/hcpol.2019.26073 PMC7020802

[30] Canada Go Justice Law Website. In:https://laws-lois.justice.gc.ca/eng/acts/C-46/section-241.2.html. Zugegriffen:

[31] Canada Go (2020) Bill C‑7: An Act to amend the Criminal Code (medical assistance in dying). https://www.justice.gc.ca/eng/csj-sjc/pl/charter-charte/c7.html

[32] Canada Go (2025) Fifth Annual Report on Medical Assistance in Dying in Canada, 2023. https://www.canada.ca/en/health-canada/services/publications/health-system-services/annual-report-medical-assistance-dying-2023.html. Accessed 23.10.2025

[33] Feichtner A, Amschl-Strablegg D (2022) Assistierter Suizid – MAiD in Kanada. In: Feichtner A, Körtner U, Likar R, Watzke H, Weixler D (eds) Assistierter Suizid: Hintergründe, Spannungsfelder und Entwicklungen. Springer Berlin Heidelberg, Berlin, Heidelberg, pp 333–340

[34] Schuklenk U, Smalling R (2017) Why medical professionals have no moral claim to conscientious objection accommodation in liberal democracies. Med Humanit 43:234–240. 10.1136/medethics-2016-103560 27106748 10.1136/medethics-2016-103560

[35] Bernier G (2020) Cost estimate for bill C‑7 „Medical Assistance in Dying“. In:The Parliamentary Budget Officer PBO. https://distribution-a617274656661637473.pbo-dpb.ca/241708b353e7782a9e5e713c2e281fc5ed932d3d07e9f5dd212e73604762bbc5. Accessed 23.10.2025

[36] Ministère de la Santé dlF, de l’Autonomie et des Personnes handicapées (2025) Mieux répondre au droit de mourir dans la dignité avec la loi Claeys-Léonetti. In: https://sante.gouv.fr/soins-et-maladies/prises-en-charge-specialisees/les-soins-palliatifs-et-la-fin-de-vie/droit-d-acces-aux-soins-palliatifs-et-a-l-accompagnement-de-la-fin-de-vie/article/mieux-repondre-au-droit-de-mourir-dans-la-dignite-avec-la-loi-claeys-leonetti. Zugegriffen:

[37] Ärzteblatt RD (2025) Frankreichs Nationalversammlung macht Weg für Recht auf Sterbehilfe frei – Deutsches Ärzteblatt. Deutsches Ärzteblatt. https://www.aerzteblatt.de/news/frankreichs-nationalversammlung-macht-weg-fur-recht-auf-sterbehilfe-frei-a1edb787-619b-4f7b-8901-677a3181caf6

[38] Parliament U (2025) Terminally Ill Adults (End of Life) Bill - Parliamentary Bills - UK Parliament. https://bills.parliament.uk/bills/3774. Accessed 23.10.2025

[39] Ärzteblatt RD (2025) Britisches Unterhaus stimmt für Legalisierung von Sterbehilfe – Deutsches Ärzteblatt. Deutsches Ärzteblatt, https://www.aerzteblatt.de/news/britisches-unterhaus-stimmt-fur-legalisierung-von-sterbehilfe-0865b1c2-1aa9-42d9-90f3-3578178b3b12

[40] Pullman D (2023) Slowing the Slide Down the Slippery Slope of Medical Assistance in Dying: Mutual Learnings for Canada and the US. AJOB Empir Bioeth 23:64–72. 10.1080/15265161.2023.2201190 37166283 10.1080/15265161.2023.2201190

[41] Raikin A (2024) From Exceptional to Routine - The Rise of Euthanasia in Canada. Cardus 2024, https://www.cardus.ca/research/health/reports/from-exceptional-to-routine/

[42] Bundesgerichtshof (2023) Beschluss in der Strafsache 4 StR 81/23. https://juris.bundesgerichtshof.de/cgi-bin/rechtsprechung/document.py?Gericht=bgh&Art=en&Datum=2023-10-25&nr=137519&anz=32&pos=6&Blank=1.pdf

[43] Föderale Kontroll- und Bewertungskommission Sterbehilfe | Volksgezondheid https://beratungsgremien.gesundheit.belgien.be/de/beratungs-und-uberlegungsgremien/foederale-kontroll-und-bewertungskommission-sterbehilfe

[44] DGPPN (2025) Eckpunkte für eine Neuregelung des assistierten Suizids – Aktualisierung 2024. https://www.dgppn.de/aktuelles/stellungnahmen-und-positionen/eckpunkte-fuer-eine-neuregelung-des-assistierten-suizids-aktualisierung-2024.html. Accessed 23.10.2025

[45] Bundesgesundheitsministerium (2025) Entwicklung, Implementierung und Evaluation eines dauerhaften Registers zur Erfassung und Analyse assistierter Suizide (RegAS). https://www.bundesgesundheitsministerium.de/ministerium/ressortforschung/handlungsfelder/forschungsschwerpunkte/suizidpraevention-staerken/regas.html. Accessed 23.10.2025

[46] Creative Commons Attribution-NonCommercial-NoDerivatives 4.0 International CC BY-NC-ND 4.0 https://creativecommons.org/licenses/by-nc-nd/4.0/deed.en

